# Anticoagulation Management Practices and Outcomes in Elderly Patients with Acute Venous Thromboembolism: A Clinical Research Study

**DOI:** 10.1371/journal.pone.0148348

**Published:** 2016-02-23

**Authors:** Charlène Insam, Marie Méan, Andreas Limacher, Anne Angelillo-Scherrer, Markus Aschwanden, Martin Banyai, Juerg- Hans Beer, Henri Bounameaux, Michael Egloff, Beat Frauchiger, Marc Husmann, Nils Kucher, Bernhard Lämmle, Christian Matter, Joseph Osterwalder, Marc Righini, Daniel Staub, Nicolas Rodondi, Drahomir Aujesky

**Affiliations:** 1 Department of General Internal Medicine, Bern University Hospital, Bern, Switzerland; 2 Clinical Trial Unit Bern, Department of Clinical Research and Institute of Social and Preventive Medicine, University of Bern, Bern, Switzerland; 3 University Clinic of Hematology and Hematologic Central Laboratory, Bern University Hospital, Bern, Switzerland; 4 Center for Thrombosis and Hemostasis, University Medical Center, Mainz, Germany; 5 Department of Angiology, Basel University Hospital, Basel, Switzerland; 6 Division of Angiology, Cantonal Hospital of Lucerne, Lucerne, Switzerland; 7 Department of Internal Medicine, Cantonal Hospital of Baden, Baden, Switzerland; 8 Division of Angiology and Hemostasis, Geneva University Hospital, Geneva, Switzerland; 9 Division of Diabetology, Geneva University Hospital, Geneva, Switzerland; 10 Department of Internal Medicine, Cantonal Hospital of Frauenfeld, Frauenfeld, Switzerland; 11 Department of Angiology, Zurich University Hospital, Zurich, Switzerland; 12 Division of Angiology, Bern University Hospital, Bern, Switzerland; 13 Cardiovascular Research, Institute of Physiology, Zurich Center for Integrative Human Physiology, University of Zurich, Zurich, Switzerland; 14 Emergency Department, Cantonal Hospital of St. Gallen, St. Gallen, Switzerland; Ottawa Hospital Research Institute, CANADA

## Abstract

Whether anticoagulation management practices are associated with improved outcomes in elderly patients with acute venous thromboembolism (VTE) is uncertain. Thus, we aimed to examine whether practices recommended by the American College of Chest Physicians guidelines are associated with outcomes in elderly patients with VTE. We studied 991 patients aged ≥65 years with acute VTE in a Swiss prospective multicenter cohort study and assessed the adherence to four management practices: parenteral anticoagulation ≥5 days, INR ≥2.0 for ≥24 hours before stopping parenteral anticoagulation, early start with vitamin K antagonists (VKA) ≤24 hours of VTE diagnosis, and the use of low-molecular-weight heparin (LMWH) or fondaparinux. The outcomes were all-cause mortality, VTE recurrence, and major bleeding at 6 months, and the length of hospital stay (LOS). We used Cox regression and lognormal survival models, adjusting for patient characteristics. Overall, 9% of patients died, 3% had VTE recurrence, and 7% major bleeding. Early start with VKA was associated with a lower risk of major bleeding (adjusted hazard ratio 0.37, 95% CI 0.20–0.71). Early start with VKA (adjusted time ratio [TR] 0.77, 95% CI 0.69–0.86) and use of LMWH/fondaparinux (adjusted TR 0.87, 95% CI 0.78–0.97) were associated with a shorter LOS. An INR ≥2.0 for ≥24 hours before stopping parenteral anticoagulants was associated with a longer LOS (adjusted TR 1.2, 95% CI 1.08–1.33). In elderly patients with VTE, the adherence to recommended anticoagulation management practices showed mixed results. In conclusion, only early start with VKA and use of parenteral LMWH/fondaparinux were associated with better outcomes.

## Introduction

The American College of Chest Physicians (ACCP) regularly issues methodologically rigorous, evidence-based clinical practice guidelines on antithrombotic therapy for acute venous thromboembolism (VTE) [1]. In these guidelines, several anticoagulation management practices, which have the potential to improve medical outcomes and to reduce the length of hospital stay (LOS), are recommended [1]. These practices include the administration of parenteral anticoagulation for ≥5 days and the achievement of an international normalized ratio (INR) ≥2.0 for ≥24 hours before stopping parenteral anticoagulation, an early start with oral vitamin K antagonists (VKA), and the initial treatment with subcutaneous low-molecular-weight heparin (LMWH) rather than with intravenous unfractionated heparin. These practices were shown to reduce the incidence of medical complications, such as death, recurrent VTE, major bleeding, thrombocytopenia and infusion phlebitis, and to decrease the LOS [2–5].

However, although elderly patients have a higher incidence of VTE and VTE-related complications than younger patients, elderly patients are underrepresented in prospective studies of VTE treatment [6–8]. Moreover, to our knowledge, whether anticoagulation management practices recommended by the ACCP guidelines are associated with improved outcomes in elderly patients with acute VTE has never been specifically assessed. In a large, prospective multicenter cohort study, we therefore examined the association between recommended anticoagulation management practices and short-term medical outcomes and LOS in elderly patients with acute VTE.

## Methods

### Cohort sample

This observational study was conducted between September 2009 and March 2012 as part of the Swiss Cohort of Elderly Patients with Venous Thromboembolism (SWITCO65+), a prospective multicenter cohort study that assessed long-term medical outcomes and quality of life in elderly patients with acute VTE. Consecutive patients aged ≥65 years with an acute, objectively confirmed VTE were identified in the inpatient and outpatient services of all five university and four high-volume non-university hospitals in Switzerland. The management of VTE, including type and duration of anticoagulation, was left entirely to the discretion of the managing physicians. Anticoagulation monitoring was done by primary care physicians, as it is common practice in Switzerland. A detailed description of the study methods was previously published [9]. The Institutional Review Board at each participating study site approved the study and patients gave written consent to participation. The approving ethic committees were the “Commission cantonale d’éthique de la recherche sur l’être humain Vaud” (site of Lausanne), “Commission cantonale d'éthique de la recherche Genève” (site of Geneva), “Kantonale Ethikkommission Bern” (site of Bern), “Kantonale Ethikkommission Zürich” (site of Zurich), “Ethikkommission Nordwest- und Zentralschweiz” (sites of Basel, Lucerne and Baden), “Ethikkommission des Kantons Thurgau” (site of Frauenfeld) and “Ethikkommission des Kantons St. Gallen” (site of St. Gallen).

### Baseline data collection

For all enrolled patients, trained study nurses prospectively collected baseline demographic information (age and sex), weight, height, comorbid conditions (active cancer, recent immobilization, chronic lung disease, heart failure, neurologic disease, history of major bleeding and VTE, and date and type of VTE), vital signs, laboratory findings (hemoglobin, serum creatinine), concomitant antiplatelet therapy, and VTE-related treatments using standardized data collection forms. VTE-related treatment information included the start and stop times/dates and the generic names of parenteral anticoagulants and VKA, insertion of a vena cava filter, systemic and catheter-based thrombolysis, and surgical thromboembolectomy.

### Anticoagulation management practices

Because our patient sample was enrolled between 2009 and early 2012, we prospectively collected anticoagulation management practices recommended by the 2008 version of the ACCP guidelines [1], including (1) administration of parenteral anticoagulants for ≥5 days; (2) achievement of an INR ≥2.0 for ≥24 hours before stopping parenteral anticoagulation; (3) start with VKA on the first treatment day (within 24 hours of VTE diagnosis); and (4) initial treatment with subcutaneous LMWH rather than with intravenous unfractionated heparin. All four practices received a strong recommendation (Grade 1) by the 2008 ACCP guidelines. We defined start with VKA on the first treatment day as the start with VKA treatment within 24 hours of VTE diagnosis. Because fondaparinux is administered once daily by subcutaneous injection, the small minority of patients who received fondaparinux as the initial treatment were grouped together with patients who received LMWH.

### Study outcomes

The study outcomes were all-cause mortality, recurrent VTE (symptomatic or fatal), and major bleeding within six months of VTE diagnosis, as done in previous studies of VTE-related quality of care [10, 11]. We defined recurrent symptomatic VTE as acute chest pain, new or worsening dyspnea or cough, acute hemoptysis, or syncope coupled with an objective diagnosis of pulmonary embolism based on spiral computed tomography, pulmonary angiography, or autopsy [12], or a new unilateral leg pain or swelling coupled with an objective diagnosis of deep vein thrombosis based on ultrasonography or contrast venography [13]. Fatal recurrent VTE was defined as death possibly or definitely related to a recurrent PE. We defined major bleeding as fatal bleeding, bleeding in a critical site or organ (intracranial, intra-spinal, intraocular, retroperitoneal, intra-articular, pericardial, or intramuscular with compartment syndrome), bleeding with reduction of hemoglobin ≥20 g/L or leading to the transfusion of ≥2 units of packed red blood cells [14]. Among patients who developed the index VTE in the outpatient setting and who were admitted to the hospital, we also recorded the LOS.

Follow-up included a surveillance face-to-face evaluation at three months and a telephone interview at six months of study participation, as well as periodic reviews of the hospital charts [9]. During each contact, study nurses interviewed patients or proxies to obtain information about mortality, VTE recurrence, and bleeding. If a clinical event had occurred, supplemental information was obtained by reviewing medical charts and interviewing the patient’s primary care physician and/or family members. A committee of three blinded clinical experts adjudicated these events. Final classifications were made on the basis of the full consensus of this committee [9].

### Statistical analyses

We presented patient baseline characteristics and the adherence to recommended anticoagulation practices as numbers and percentages or medians and interquartile ranges, as appropriate. We examined the association between anticoagulation practices and the time to death, a first VTE recurrence and a first major bleeding within six months of the index VTE using Cox proportional hazard models. For each model, we adjusted for selected variables that have previously been found to be associated with the specific outcome, i.e., short-term all-cause mortality [15–17], major bleeding [15, 18–20], and VTE recurrence [15, 21–24]. Because anticoagulation practices usually differ in patients who receive invasive treatments, i.e. thrombolysis, a vena cava filter, or surgical thromboembolectomy, such patients were excluded from all analyses. When analyzing practices pertaining to the overlap of parenteral anticoagulants and VKA (parenteral anticoagulation for ≥5 days, INR ≥2.0 for ≥24 hours, and start with VKA on the first treatment day), we also excluded patients who received monotherapy with parenteral anticoagulants (e.g., patients with cancer) or VKA, no anticoagulation at all, and those in whom the index VTE occurred under therapeutic anticoagulation. Similarly, when analyzing the use of LMWH/fondaparinux as the initial treatment, we excluded patients with severe renal failure (glomerular filtration rate <30 ml/min./m^2^) because unfractionated heparin may be the treatment of choice in such patients [1].

In the subset of outpatients with VTE who were admitted to the hospital, we assessed the association between anticoagulation practices and the LOS using a lognormal survival model, adjusting for a broad set of patient baseline characteristics. The model yields time ratios (TR), with a TR above 1 indicating a prolonged LOS and a TR below 1 a shortened LOS.

Given the low proportion of missing values except for arterial oxygen saturation (23%), we assumed missing values to be normal. When we used multiple imputation for the missing values of arterial oxygen saturation, the adjusted hazard rates and the time ratio remained almost identical. All analyses were done using Stata 13 (Stata Corporation, College Station, Texas).

## Results

### Study sample

Of the 1003 patients enrolled in the cohort [9], we excluded 12 patients who did not allow the use of their data or withdrew from the study within one day, leaving a final sample of 991 analyzed patients. One patient was lost to follow up, 26 patient withdrew consent within 6 months but were included in the analysis. The median age was 75 years, 47% of patients were women, 63% had VTE in the outpatient setting and were admitted to the hospital, 69% had symptomatic pulmonary embolism with or without deep vein thrombosis, 18% active cancer, and 6% severe renal failure (Table 1). Overall, 4% (41/991) of patients received invasive treatments (30 systemic or catheter based-thrombolysis, 11 vena cava filter insertions, and/or 3 surgical thromboembolectomy). The median duration of anticoagulation was 8 months (interquartile range 4 to 24 months).

**Table 1:** Patient baseline characteristics (N = 991).

Characteristic	Median (IQR) or n (%)^a^	
Age, years	75	(69–81)
Female sex	463	(47)
Outpatients with VTE who were admitted to the hospital	621	(63)
Body mass index >30 kg/m^2^	237	(24)
Active cancer^b^	178	(18)
Recent immobilization^c^	219	(22)
Chronic lung disease^d^	136	(14)
Heart failure^e^	115	(12)
Neurologic disease with hemiparesis, hemiplegia, or paraplegia	29	(3)
History of major bleeding	101	(10)
History of VTE	283	(29)
Clinically overt pulmonary embolism	687	(69)
Unprovoked VTE^f^	694	(70)
Pulse rate ≥110 beats/minute	88	(9)
Systolic blood pressure <100 mm Hg	35	(4)
Arterial oxygen saturation <90%^g^	107	(11)
Hemoglobin <130 g/L for men and <120 g/L for women	388	(39)
Creatinine >107 μmol/L	226	(23)
Severe renal failure^h^	55	(6)
Concomitant antiplatelet therapy^i^	321	(32)
Index VTE occurred under therapeutic anticoagulation^j^	51	(5)
Initial treatment with parenteral anticoagulants	957	(97)
LMWH^k^	465	(47)
Fondaparinux	158	(16)
Unfractionated heparin	333	(34)
Danaparoid	1	(0.1)
No parenteral anticoagulation	34	(3)
Systemic or catheter-based thrombolysis	30	(3)
Vena cava filter insertion	11	(1)
Surgical thrombembolectomy	3	(0.3)

Abbreviations: IQR = interquartile range; VTE = venous thromboembolism; LMWH = low-molecular-weight heparin.

^a^Missing values were 0.5% for body mass index, 0.1% for history of major bleeding, 6% for hemoglobin, 8% for creatinine, 2% for pulse rate, 2% for systolic blood pressure, 23% for arterial oxygen saturation.

^b^Chemotherapy, radiotherapy, surgery, and/or palliative care during the last 3 months.

^c^Bed rest >72 hours, voyage in a sitting position for >6 hours, or fracture or a cast of the lower extremity during the last 3 months.

^d^Chronic obstructive pulmonary disease, active asthma, lung fibrosis, cystic fibrosis, or bronchiectasis.

^e^Systolic/diastolic heart failure, left/right heart failure, forward or backward heart failure, known left ventricular ejection fraction of <40%, or acute heart failure NYHA III/IV during the last 3 months.

^f^Occurrence of VTE in the absence of estrogen therapy, major surgery, or immobilization during the last 3 months.

^g^With or without the administration of supplemental oxygen.

^h^Severe renal failure defined by a glomerular filtration rate <30 ml/minute/m^2^.

^i^Aspirin, clopidogrel, prasugrel, and/or dipyridamol.

^j^Therapeutic anticoagulation with vitamin K antagonists or full-dose parenteral anticoagulation.

^k^Dalteparin, enoxaparin, or nadroparin.

### Adherence to recommended anticoagulation management practices

Among patients who received parenteral anticoagulation with concomitant VKA, the adherence to our pre-defined anticoagulation practices was very variable (Table 2): 86% (667/774) received parenteral anticoagulation ≥5 days, 36% (276/774) achieved an INR ≥2.0 for ≥24 hours before parenteral anticoagulation was stopped, and in 54% (414/774) VKA therapy was started within 24 hours of VTE diagnosis. Overall, 66% (589/898) of patients received LMWH or fondaparinux as the initial parenteral anticoagulant.

**Table 2:** Adherence to anticoagulation management practices.

Anticoagulation practice	n/N	(%)
Parenteral AC ≥5 days	667/774^a^	(86)
INR ≥2.0 for ≥24 hours before stopping parenteral AC	276/774^a^	(36)
Start with VKA on the first treatment day	414/774^a^	(54)
Initial treatment with LMWH or fondaparinux	589/898^b^	(66)

Abbreviations: AC = anticoagulation; INR = international normalized ratio; VKA = vitamin K antagonist; LMWH = low-molecular-weight heparin.

^a^Patients receiving parenteral anticoagulant monotherapy (n = 122), VKA monotherapy (n = 25), no AC (n = 9), vena cava filter insertion (n = 11), systemic or catheter-based thrombolysis (n = 30), surgical thromboembolectomy (n = 3), or in whom the index venous thromboembolism occurred under therapeutic AC (n = 51) were excluded. Patients could have more than one exclusion criterion.

^b^Patients with vena cava filter insertion (n = 11), systemic or catheter-based thrombolysis (n = 30), surgical thromboembolectomy (n = 3), or a glomerular filtration rate <30 ml/minute/m^2^ (n = 55) were excluded. Patients could have more than one exclusion criterion.

### Association between anticoagulation management practices and outcomes

Within six months of VTE diagnosis, 9% (85/991) of patients died, 3% (28/991) had recurrent VTE, and 7% (70/991) had major bleeding. Overall, 2% of patients had fatal PE (20/991) and fatal bleeding (19/991), respectively. The median LOS was 8 days (interquartile range, 5–11 days). After adjustment, no anticoagulation practice was associated with mortality or VTE recurrence at six months (Table 3). However, start with VKA on the first treatment day was associated with a lower risk of major bleeding (adjusted hazard ratio [HR] 0.37, 95% confidence interval [CI] 0.20–0.71) (Table 3). In patients with early vs. late start of VKA, 47% of major bleeds occurred during the first treatment month, and 38% of bleeds occurred under parenteral anticoagulation. The risk of major bleeding did not differ by type of parenteral anticoagulant used (LMWH/fondaparinux vs.unfractionated heparin) or the site of treatment (home vs. hospital) in these patients. The percentage of time in the therapeutic INR range did not differ between patients with early vs. late start of VKA treatment (58 vs%. 56%; P = 0.28).

**Table 3:** Associations between anticoagulation practices and clinical outcomes at 6 months.

	Adherence n/N (%)			
	Yes	No		(95% CI)
**Mortality**			Adjusted HR*	
Parenteral AC ≥5 days	34/667 (5.1)	4/107 (3.7)	1.14	(0.39–3.27)
INR ≥2.0 for ≥24 hours before stopping parenteral AC	15/276 (5.4)	23/498 (4.6)	0.75	(0.37–1.53)
Start with VKA on the first treatment day	11/414 (2.7)	27/360 (7.5)	0.49	(0.23–1.03)
Initial treatment with LMWH or fondaparinux	40/589 (6.8)	30/309 (9.7)	0.83	(0.51–1.34)
**Recurrent venous thromboembolism**			**Adjusted HR**†	
Parenteral AC ≥5 days	17/667 (2.5)	3/107 (2.8)	0.92	(0.27–3.14)
INR ≥2.0 for ≥24 hours before stopping parenteral AC	7/276 (2.5)	13/498 (2.6)	0.96	(0.38–2.42)
Start with VKA on the first treatment day	8/414 (1.9)	12/360 (3.3)	0.52	(0.21–1.28)
Initial treatment with LMWH or fondaparinux	14/589 (2.4)	10/309 (3.2)	0.72	(0.32–1.62)
**Major bleeding**			**Adjusted HR**‡	
Parenteral AC ≥5 days	39/667 (5.8)	6/107 (5.6)	1.02	(0.43–2.42)
INR ≥2.0 for ≥24 hours before stopping parenteral AC	15/276 (5.4)	30/498 (6.0)	0.88	(0.47–1.64)
Start with VKA on the first treatment day	14/414 (3.4)	31/360 (8.6)	0.37	(0.20–0.71)
Initial treatment with LMWH or fondaparinux	34/589 (5.8)	25/309 (8.1)	0.78	(0.46–1.33)

Abbreviations: AC = anticoagulation; HR = hazard ratio; CI = confidence interval; INR = international normalized ratio; VKA = vitamin K antagonist; LMWH = low-molecular-weight heparin.

*Adjusted for age, sex, active cancer, chronic lung disease, heart failure, history of major bleeding, recent immobilization, clinically overt pulmonary embolism, pulse rate ≥110 beats/minute, systolic blood pressure <100 mm Hg, and arterial oxygen saturation <90%.

^†^Adjusted for age, cancer, history of venous thromboembolism, and type of venous thromboembolism (unprovoked vs. provoked).

^‡^Adjusted>Adjusted for age, cancer, history of major bleeding, clinically overt pulmonary embolism, hemoglobin level, creatinine level, and concomitant antiplatelet therapy.

Whereas start with VKA on the first treatment day (adjusted TR 0.77, 95% CI 0.69–0.86) and initial treatment with LMWH or fondaparinux (adjusted TR 0.87, 95% CI 0.78–0.97) were associated with a decreased LOS, the achievement of an INR ≥2.0 for ≥24 hours before stopping parenteral anticoagulation was associated with an increased LOS (adjusted TR 1.20, 95% CI 1.08–1.33) (Table 4). Patients who received parenteral anticoagulants ≥5 days were also more likely to have a prolonged LOS but the association failed to achieve statistical significance (adjusted TR 1.14, 95% CI 0.99–1.32).

**Table 4:** Association between anticoagulation practices and length of hospital stay*.

	Practice performed			
	Yes	No		
Anticoagulation practice	Median LOS		Time ratio†	(95% CI)
Parenteral AC ≥5 days	8.0	6.0	1.14	(0.99–1.32)
INR ≥2.0 for ≥24 hours before stopping parenteral AC	8.0	7.0	1.20	(1.08–1.33)
Start with VKA on the first treatment day	6.0	8.0	0.77	(0.69–0.86)
Initial treatment with LMWH or fondaparinux	7.0	8.0	0.87	(0.78–0.97)

Abbreviations AC = anticoagulation; LOS = length of hospital stay; CI = confidence interval; INR = international normalized ratio; LMWH = low-molecular-weight heparin.

*Subgroup of outpatients with venous thromboembolism who were admitted to the hospital (n = 621).

^†^Adjusted for age, sex, body mass index, active cancer, chronic lung disease, heart failure, neurologic disease, history of major bleeding, history of venous thromboembolism, recent immobilization, pulse rate ≥110 beats/minute, systolic blood pressure <100 mm Hg, arterial oxygen saturation <90%, hemoglobin level, creatinine level, concomitant antiplatelet therapy, unprovoked venous thromboembolism, and clinically overt pulmonary embolism

When analyzing practices pertaining to the overlap of parenteral anticoagulants and VKA (parenteral anticoagulation for ≥5 days, INR ≥2.0 for ≥24 hours, and start with VKA on the first treatment day), we excluded patients who received monotherapy with parenteral anticoagulants (e.g., patients with cancer) or VKA, no anticoagulation at all, and those in whom the index VTE occurred under therapeutic anticoagulation. Overall, only 90 (51%) of 178 patients with cancer received VKA treatment. When these patients were excluded from analysis, our results did not change markedly.

## Discussion

In our prospective multicenter cohort study of elderly patients with acute VTE, the adherence to the four recommended anticoagulation management practices was highly variable (36–86%). Overall, two out of four practices were associated with a lower rate of major bleeding and/or a shorter LOS.

As expected, starting VKA on the first treatment day was associated with a reduction of the median LOS by two days. These findings are consistent with a meta-analysis of five randomized controlled trials in which hospital stay was approximately four days shorter in patients with an early start of VKA [25]. In our study, early start with VKA was also associated with a significant decrease in major bleeding. In a prior meta-analysis, early initiation with VKA was associated with a lower risk of minor but not major bleeding [25]. The beneficial effect of starting VKA early on incidence of minor bleeding has been explained by concomitant earlier withdrawal of heparin, which shortens the exposure to this antithrombotic agent [25]. In a retrospective study of U.S. veterans, the administration of warfarin within one day of starting heparin was associated with a significantly lower overall 90-day complication rate (death, VTE recurrence, or major bleeding) [26]. However, the lower bleeding rate in patients with an early VKA start could also be the effect of an indication bias, i.e. physicians may deliberately delay VKA treatment in patients perceived to have a higher bleeding risk. A prior study demonstrated that patients with an early start with VKA also had a lower risk of thrombocytopenia and heparin-induced infusion phlebitis [4]. Overall, these results confirm that an early start of VKA has the potential to safely reduce costly hospital days in elderly patients with VTE. The fact that only half of patients received early VKA treatment in our study suggests that this cost-saving anticoagulation practice is still underused in elderly patients with acute VTE.

The 2008 ACCP guidelines recommend parenteral anticoagulant treatment ≥5 days and the achievement an INR ≥2.0 for ≥24 hours before stopping parenteral anticoagulation because randomized trials of VTE treatment often used heparin therapy for a minimum duration of five days [1, 27–29]. Moreover, there is evidence from animal studies that VKA require several days before developing a therapeutic antithrombotic effect [30, 31]. While the adherence to these anticoagulation practices was not associated with improved clinical outcomes, both practices were associated with a 1 to 2-day increase in LOS. This does not come as a surprise because physicians may be reluctant to discharge elderly patients before the 5-day course of parenteral anticoagulation is completed and patients are successfully switched to VKA treatment within the therapeutic range. Further studies should examine whether a shorter course of parenteral anticoagulants (e.g., three days) is as effective as a 5-day course. Care organizations that facilitate the switch from parenteral anticoagulants to VKA in the outpatient setting may have the potential to substantially decrease the LOS.

Compared to intravenous unfractionated heparin, initial parenteral anticoagulation with subcutaneous LMWH/fondaparinux was associated with shorter LOS because the latter treatment can be given on an outpatient basis [10, 12]. In our study, the use of LMWH/fondaparinux reduced the median LOS by one day. A meta-analysis of randomized controlled trials demonstrated that LMWH was associated with significantly fewer deaths (odds ratio, 0.76) and lower rates of recurrent VTE (odds ratio, 0.68) and major bleeding (odds ratio, 0.57) [2]. While our study did not find any association between use of LMWH/fondaparinux and clinical outcomes, it may not have enough power to detect such an association. In our study, only about two-thirds of elderly patients without severe renal failure received LMWH/fondaparinux. Given its proven benefit to reduce the LOS and its potential to improve patient outcomes, the use of parenteral anticoagulation with LMWH or fondaparinux should be encouraged in elderly patients with acute VTE.

Our study has potential limitations. First, because new oral anticoagulants (direct thrombin and factor Xa inhibitors) were not authorized for the treatment of acute VTE in Switzerland at the time of patient enrollment, we could not evaluate the potential impact of these drugs on patient outcomes. Second, we could not study the association between anticoagulation practices and other clinically relevant outcomes, such as heparin-induced thrombocytopenia or infusion phlebitis. Third, because we had only a limited number of VTE recurrences (n = 28) during follow-up, our study may have been underpowered to detect weaker associations between anticoagulation practices and VTE recurrence. Fourth, despite extensive adjustment, the observed outcome differences may be attributable to confounding related to unmeasured severity of illness. Thus, regarding the association of early start with VKA and lower major bleeding risk, there might be a patients selection process, where physicians tend to start oral anticoagulation early in healthier patients who have a lower bleeding risk. Finally, because we focused on initial anticoagulation practices, we cannot exclude the possibility that other anticoagulation-related factors (e.g., anticoagulation quality) and treatments during follow-up have influenced medical outcomes.

## Conclusion

In conclusion, the adherence to recommended anticoagulation management practices showed mixed results in elderly patients with VTE. Only early start with VKA and use of parenteral LMWH/fondaparinux rather than unfractionated heparin were associated with better outcomes. Given the suboptimal adherence rate of these practices and their potential clinical and economic benefit, their implementation could be useful.
